# ZNF469 drives TGF-β1/SMAD3-mediated extracellular matrix regulation in pulmonary fibrosis

**DOI:** 10.17305/bb.2026.14165

**Published:** 2026-05-28

**Authors:** Thammachanok Boonto, Nucharnart Suntornnont, Archittapon Nokkeaw, Chaiyaboot Ariyachet, Amornpun Wongkarnjana

**Affiliations:** 1 Department of Biochemistry, Faculty of Medicine, Chulalongkorn University, Bangkok, Thailand; 2 Center of Excellence in Hepatitis and Liver Cancer, Faculty of Medicine, Chulalongkorn University, Bangkok, Thailand; 3 Division of Pulmonology and Critical Care Medicine, Department of Medicine, Faculty of Medicine, Chulalongkorn University, Bangkok, Thailand; 4 Division of Pulmonology and Critical Care Medicine, Department of Medicine, King Chulalongkorn Memorial Hospital, Thai Red Cross Society, Bangkok, Thailand

**Keywords:** Lung fibroblast, pulmonary fibrosis, extracellular matrix, transcription factor, transforming growth factor-β

## Abstract

Pulmonary fibrosis is characterized by persistent activation of lung fibroblasts and excessive extracellular matrix (ECM) deposition; however, the transcriptional mechanisms sustaining this activated state remain poorly understood. Given the established profibrotic role of zinc finger protein 469 (ZNF469) in other mesenchymal lineages, we investigated its function in lung fibroblasts using an integrated functional and transcriptomic approach. We found that *ZNF469* depletion attenuated key features of fibroblast activation, including proliferation, migration, and contractile capacity, concomitantly reducing collagen production and deposition. Mechanistically, combined transcriptomic profiling and Cleavage Under Targets and Release Using Nuclease (CUT&RUN) assays revealed that ZNF469 modulates ECM-associated transcriptional regulation, demonstrating preferential occupancy at the promoters of key collagen genes, such as collagen type I alpha 1 chain (*COL1A1*) and collagen type III alpha 1 chain (*COL3A1*). Furthermore, we demonstrated that transforming growth factor-β1 (TGF-β1) stimulated ZNF469 expression in a SMAD family member 3 (SMAD3)-dependent manner, with CUT&RUN further revealing SMAD3 occupancy at the *ZNF469* promoter. Importantly, ZNF469 depletion blunted TGF-β1-induced collagen production, indicating that ZNF469 contributes to the profibrotic effects of the TGF-β1/SMAD3 axis. Finally, we underscored the clinical relevance of ZNF469 by analyzing bulk and single-cell RNA sequencing data from patients with idiopathic pulmonary fibrosis and systemic sclerosis. *ZNF469* was found to be upregulated in fibrotic lung tissues, particularly within the activated fibroblast lineage, and correlated with key ECM gene signatures. Our findings collectively support a model wherein the TGF-β1/SMAD3/ZNF469 axis contributes significantly to ECM gene regulation and fibroblast activation, thereby positioning ZNF469 as a critical profibrotic factor and a promising therapeutic target in pulmonary fibrosis.

## Introduction

The extracellular matrix (ECM) is a sophisticated network comprising numerous structural and regulatory components, including collagens, proteoglycans, and glycoproteins. This matrix provides structural support and transduces biomechanical signals that regulate cellular function and identity [1, 2]. The composition of the ECM is continuously influenced by precisely coordinated processes of synthesis, degradation, and remodeling, which are vital for maintaining tissue homeostasis [3]. This dynamic regulation governs ECM quantity, structure, and composition, facilitating the release of essential signaling molecules [4]. As a result, ECM remodeling must be meticulously regulated to preserve tissue integrity and homeostasis.

Pulmonary fibrosis is a chronic lung disease characterized by excessive deposition of ECM components, leading to progressive architectural distortion and impaired respiratory function [5]. Growing evidence suggests that pulmonary fibrosis results from maladaptive activation of lung fibroblasts, which adopt a hyperproliferative and profibrotic phenotype [5]. Repetitive epithelial injury, triggered by environmental factors such as exposure to toxins, smoking, viral infections, aging, or genetic predisposition, is widely recognized as a key initiating event that promotes sustained fibroblast activation and excessive matrix production, ultimately disrupting ECM homeostasis [6, 7]. While fibroblasts are the predominant matrix-producing cells in fibrosis, their activation is also influenced by dynamic interactions with adjacent lung-resident cell populations. Alveolar epithelial type 2 cells, crucial regulators of alveolar homeostasis and epithelial renewal, significantly impact fibroblast behavior [8, 9]. Under chronic stress, epithelial cells undergo functional reprogramming and secrete profibrotic mediators such as transforming growth factor-β1 (TGF-β1), thereby enhancing fibroblast proliferation and myofibroblast differentiation, ultimately driving pulmonary fibrosis [10, 11].

Idiopathic pulmonary fibrosis (IPF) is the most common and clinically significant form of pulmonary fibrosis, characterized by an unknown cause. Despite advancements in clinical management, untreated IPF has a median survival of only 3–5 years, with mortality rates comparable to or exceeding those of several common cancers [12, 13]. Although antifibrotic therapies such as nintedanib and pirfenidone have shown modest efficacy, they do not reverse established fibrosis and are often limited by tolerability [5]. These challenges underscore the necessity of a deeper understanding of the molecular mechanisms driving fibrogenesis to facilitate the development of more targeted and transformative therapies.

Cellular identity and behavior are shaped not only by genomic sequence but also by dynamic gene regulatory programs. Transcription factors orchestrate these programs by binding to promoter regions and distal regulatory elements, integrating signals from the cellular environment to control context-dependent gene expression [14, 15]. In the context of fibrosis, aberrant activation of key transcription factors establishes self-sustaining transcriptional programs that maintain fibroblasts in a hyperactive, ECM-producing state under pathological conditions [16]. Despite increasing recognition of this paradigm, the identity and hierarchical organization of ECM-specific transcriptional regulators in the fibrotic state remain incompletely characterized.

Zinc finger protein 469 (ZNF469) was initially identified as a causative gene in brittle cornea syndrome (BCS), a connective tissue disorder characterized by corneal thinning and progressive visual impairment [17, 18]. Recurrent frameshift mutations in *ZNF469* have been documented to disrupt collagen integrity and connective tissue architecture across diverse populations [17–19]. Due to its partial sequence homology with collagen genes, ZNF469 has been hypothesized to regulate collagen synthesis and ECM organization [18]. Recent groundbreaking studies have identified ZNF469 as a key transcription factor that enhances the expression of collagen and various ECM genes across multiple tissues [20–22]. In dermal fibroblasts, keloid fibroblasts, and hepatic stellate cells, ZNF469 knockdown significantly impairs proliferation, migration, and contractile capacity. Mechanistically, ZNF469 binds to regulatory regions of collagen and ECM-associated genes [20–22]. Cross-species studies further support its structural role, as deletion of the *ZNF469* ortholog in mice and zebrafish disrupts ECM composition and tissue integrity [23, 24]. While its role in the liver and skin is increasingly elucidated, the function of ZNF469 in pulmonary fibrosis remains unexplored.

In this study, we aim to characterize the role of ZNF469 in lung fibroblast activation and pulmonary fibrosis. Utilizing an integrated approach that combines functional assays, bulk and single-cell transcriptomics, and cleavage under targets and release using nuclease (CUT&RUN) profiling, we provide evidence that ZNF469 contributes to ECM production and acts as a downstream mediator of the TGF-β1/SMAD family member 3 (SMAD3) signaling axis in lung fibroblasts. Furthermore, our clinical findings reveal that *ZNF469* is significantly upregulated in human fibrotic lung tissues, particularly within profibrotic fibroblast subpopulations, and positively correlates with ECM gene expression signatures, highlighting its potential as a therapeutic target for pulmonary fibrosis.

## Materials and methods

### Cell culture and generation of lentiviral particles

Human lung fibroblast cell lines IMR-90 (cat. no. CCL-186) and MRC-5 (cat. no. CCL-171) were obtained from the American Type Culture Collection (ATCC, Manassas, VA, USA) and maintained in Minimum Essential Medium (cat. no. 11095080; Thermo Fisher Scientific, Carlsbad, CA, USA) supplemented with 10% fetal bovine serum (FBS) (cat. no. A5256701; Thermo Fisher Scientific), 100 µg/mL Primocin (cat. no. ant-pm-05; InvivoGen, San Diego, CA, USA), 1% Antibiotic-Antimycotic (cat. no. 15240062; Thermo Fisher Scientific), and GlutaMAX (cat. no. 35050061; Thermo Fisher Scientific). Cultures were maintained at 37 ^∘^C in a humidified incubator with 5% CO2. HEK293FT cells (cat. no. R70007; Thermo Fisher Scientific) served as host cells for lentiviral production and were cultured under identical conditions, with Dulbecco’s Modified Eagle Medium (cat. no. 11965–092; Thermo Fisher Scientific) as the basal medium.

To generate *ZNF469* and *SMAD3* knockdown cell lines, two independent short hairpin RNA (shRNA) sequences (designated as sh-1 and sh-2 for each target) targeting human *ZNF469* and *SMAD3* transcripts were designed using the Genetic Perturbation Platform (GPP; Broad Institute, Cambridge, MA, USA). Oligonucleotides encoding these shRNA sequences were synthesized, annealed, and ligated into AgeI- and EcoRI-digested Tet-pLKO-puro lentiviral vectors (cat. no. 21915; Addgene, Watertown, MA, USA). All constructs were verified by Sanger sequencing. Cloning primer sequences are provided in Table S1. Lentiviral particles were produced in HEK293FT cells by co-transfecting shRNA-containing Tet-pLKO-puro plasmids, psPAX2 (cat. no. 12260; Addgene), and pMD2.G (cat. no. 12259; Addgene) at a ratio of 3.08:3.08:2 µg per well of a six-well plate using Lipofectamine 3000 (cat. no. L3000015; Thermo Fisher Scientific). Viral supernatants were collected and filtered prior to transduction by spinoculation (1,000 × g, 40 min, 37 ^∘^C), as previously described [25]. The stable inducible lines were selected using 0.5 µg/mL puromycin (cat. no. P8833; Sigma-Aldrich, St. Louis, MO, USA). Induction of short hairpins was achieved by treating cells with 2 µg/mL doxycycline (cat. no. D5207; Sigma-Aldrich) for 4 days before downstream assays [26]. Doxycycline-treated cells were designated as DOX+, while cells cultured under identical conditions without doxycycline served as DOX-- controls. To assess responsiveness to profibrotic signaling, cells were treated with culture medium containing 10 ng/mL TGF-β1 (cat. no. 100-21-10UG; Thermo Fisher Scientific) for 24 hours prior to downstream experiments. For mitomycin C experiments, cells were incubated with culture medium containing 10 µg/mL mitomycin C (cat. no. 50-07-7; Sigma-Aldrich) for 2 h to inhibit proliferation, followed by a phosphate-buffered saline (PBS) wash and 48 h of maintenance in fresh medium before functional assays.

### RNA extraction and reverse transcription quantitative PCR

Total RNA was isolated from cells using the GenUP Total RNA Kit (cat. no. BR0700902; Biotechrabbit, Berlin, Germany) per the manufacturer’s instructions, and cDNA was synthesized using standard reverse transcription procedures with iScript Reverse Transcription Supermix (cat. no. 1708841; Bio-Rad Laboratories, Hercules, CA, USA). Reverse transcription quantitative PCR (RT-qPCR) was performed under the following thermocycling conditions: 95 ^∘^C for 10 min, followed by 40 cycles of 95 ^∘^C for 15 s and 60 ^∘^C for 1 minute. Melt curve analysis (95 ^∘^C for 15 s, 60 ^∘^C for 1 minute, and 95 ^∘^C for 15 s) was conducted to assess amplification specificity. Relative gene expression was calculated using the comparative 2-ΔΔCq method, normalizing to ribosomal protein L19 expression, and comparisons were performed against the corresponding control condition using the statistical test indicated in the figure legend [27, 28]. Primer sequences for RT-qPCR were designed using PrimerQuest software (Integrated DNA Technologies, Coralville, IA, USA) and are listed in Table S1.

### Cell proliferation assay

Cell proliferation was assessed using an 3-(4,5-dimethylthiazol-2-yl)-2,5-diphenyltetrazolium bromide (MTT)-based colorimetric assay (cat. no. M6494; Thermo Fisher Scientific) as previously described [25]. Briefly, cells were seeded in 96-well plates at a density of 5 × 10ˆ3 cells/well in complete culture medium. At specified time points (0, 2, 4, and 6 days), MTT reagent was added to each well at a final concentration of 0.5 mg/mL in basal medium. After a 2-hour incubation at 37 ^∘^C, the medium was removed, and formazan crystals were solubilized in dimethyl sulfoxide (DMSO). Absorbance was measured at 570 nm using a Synergy HTX microplate reader (Agilent Technologies, Santa Clara, CA, USA). Background-corrected absorbance values were normalized to the day 0 reading for each corresponding condition to determine relative proliferation.

### Cell migration assay

Cell migration was evaluated using a transwell assay [29]. Cells were serum-starved for 24 h prior to seeding. Doxycycline induction, as indicated, was maintained throughout the assay duration to sustain shRNA-mediated knockdown. Following serum starvation, cells were trypsinized and seeded into the upper chamber of 8-µm pore transwell inserts (cat. no. 353097; Corning Life Sciences, Corning, NY, USA) at a density of 2.5 × 10ˆ4 cells/insert in 200 µL of serum-free medium. The lower chamber contained 600 µL of complete medium supplemented with 10% FBS to serve as a chemoattractant. After 24 h of incubation, migrated cells on the underside of the membrane were fixed with absolute methanol, stained with 0.1% crystal violet, and imaged using the EVOS M7000 imaging system (Thermo Fisher Scientific). Migrated cell counts were quantified using ImageJ software (version 1.53t; National Institutes of Health, Bethesda, MD, USA)**.** Quantification was performed by averaging counts from 10 random fields per insert across independent experiments.

### Collagen gel contraction assay

Fibroblast contractile activity was assessed using the collagen gel contraction assay. Cells were harvested, counted, and resuspended in a neutralized rat tail type I collagen solution (1 mg/mL; cat. no. C3867; Sigma-Aldrich). The collagen-cell suspension was cast into 48-well plates at a density of 5 × 10ˆ4 cells/gel in a final gel volume of 300 µL/well. Gels were allowed to polymerize at room temperature for 20 min and were subsequently detached from the well edge using a sterile P10 pipette tip to facilitate free-floating, isotropic contraction. After detachment, culture medium was gently added to each well, and gels were incubated at 37 ^∘^C with 5% CO2 for 24 h. At the conclusion of the incubation period, gels were imaged under brightfield mode using a UVP ChemStudio instrument (Analytik Jena, Jena, Germany). Gel area was quantified using ImageJ software by manually outlining the gel boundaries.

### Immunofluorescence staining

Cells were fixed in 4% paraformaldehyde (cat. no. J61899.AP; Thermo Fisher Scientific) for 15 min at room temperature, permeabilized with 0.1% Triton X-100 (cat. no. X100; Sigma-Aldrich) for 5 min, and blocked with 1% bovine serum albumin (BSA; cat. no. BSA-1S; Capricorn Scientific) for 1 hour. Primary antibodies against type I collagen (1:1,000; cat. no. 8-3A5; Developmental Studies Hybridoma Bank, Iowa City, IA, USA), ZNF469 (1:1,000; cat. no. PA5-145175; Thermo Fisher Scientific), Ki-67 (1:1,000; cat. no. ab15580; AbCam, Cambridge, MA, USA), or SMAD3 (1:1,000; cat. no. 9523; Cell Signaling Technology, Danvers, MA, USA) were incubated overnight at 4 ^∘^C. This was followed by incubation with Alexa Fluor 488-conjugated anti-mouse or anti-rabbit secondary antibodies (1:500; cat. no. A-21202 and A-21206, respectively; Thermo Fisher Scientific) at room temperature for 1 hour. Nuclei were counterstained with 300 nM 4′,6-diamidino-2-phenylindole (DAPI) (cat. no. D9542, Sigma-Aldrich).

For intracellular collagen analysis, cells were cultured for 4 days, while for extracellular collagen fibril assembly, cells were cultured for 8 days. The culture medium was supplemented with 20 µM L-ascorbic acid (cat. no. A4544; Sigma-Aldrich) to promote collagen hydroxylation and fibrillar assembly. For extracellular collagen fibril staining, cells were subsequently fixed and subjected to the previously described staining workflow, with the critical exception that the Triton X-100 permeabilization step was omitted to preserve and specifically visualize extracellular matrix-associated collagen. All images were acquired on an EVOS M7000 Imaging System using identical exposure settings to ensure quantitative comparability. The integrated fluorescence density or mean fluorescence intensity was quantified for each field and normalized to the number of DAPI-positive nuclei in the corresponding field. At least 10 randomly selected fields per condition from two independent experiments were analyzed.

### CUT&RUN assay

Promoter analysis was conducted using CUT&RUN assays with the hyperactive protein G–micrococcal nuclease (pG-MNase) CUT&RUN assay kit (cat. no. HD101; Vazyme Biotech, Nanjing, China), following the manufacturer’s protocol. In brief, 5 x 10ˆ5 cells were harvested and incubated with Concanavalin A magnetic beads in binding buffer. After gentle permeabilization, bead-bound cells were incubated overnight at 4 ^∘^C with either normal rabbit IgG (cat. no. 2729; Cell Signaling Technology) as a negative control, anti-ZNF469 antibody (cat. no. PA5-145175; Thermo Fisher Scientific), or anti-SMAD3 antibody (cat. no. 9523; Cell Signaling Technology). Following washing steps, bead-cell complexes were incubated with pG-MNase enzyme with rotation at 4 ^∘^C for 1 hour. Targeted chromatin cleavage was initiated by the addition of CaCl_2_ and allowed to proceed at 4 ^∘^C for 2 h. The reaction was terminated by adding stop buffer containing spike-in DNA for normalization. Enriched chromatin DNA was purified using FastPure gDNA mini columns supplied with the kit and subsequently analyzed by qPCR using custom-designed primers specific for the promoters of candidate collagen and ECM-related genes. CUT&RUN-qPCR signals were normalized to spike-in DNA and are presented as fold enrichment relative to the IgG negative control after normalization to input. Primer sequences were designed using PrimerQuest based on University of California, Santa Cruz (UCSC) Genome Browser annotations and are listed in Table S1.

### RNA sequencing (RNA-seq) analysis

Purified total RNA isolated from IMR-90 fibroblasts following *ZNF469* knockdown by sh-2 and the corresponding non-induced (DOX--) control was vacuum-dried in GenTegra-RNA microtubes (GenTegra, Pleasanton, CA, USA) and processed by Biomarker Technologies (Münster, Germany) for RNA-seq. Samples (*n* ═ 3 per group) were quality-checked using the Agilent Bioanalyzer 2100 system (Agilent Technologies) and subjected to poly(A)-selected library construction using the NEBNext Ultra II RNA Library Prep Kit for Illumina (cat. no. E7770; New England Biolabs, Ipswich, MA, USA). Libraries were subsequently sequenced on an Illumina NovaSeq 6000 (Illumina, San Diego, CA, USA). Clean reads with a minimum Phred quality score of 30 (Q30) and a retention rate of 90% were selected and aligned to the human reference genome (GRCh38/hg38) using HISAT2 and StringTie. Differentially expressed genes (DEGs) were defined by |log2 fold change| > 1 and a false discovery rate (FDR) < 0.05. Functional overrepresentation analyses for Kyoto Encyclopedia of Genes and Genomes (KEGG) pathways and Gene Ontology (GO) were performed using ShinyGO (version 0.81; South Dakota State University, Brookings, SD, USA) [30]. Gene Set Enrichment Analysis (GSEA; version 4.3.3; Broad Institute) was conducted using a ranked list of detected genes against curated ECM-associated terms or pathways [31, 32]. Significantly enriched pathways were defined using an FDR threshold < 0.05. Leading-edge analysis within GSEA was utilized to identify genes driving pathway enrichment, which were subsequently validated via RT-qPCR analysis.

### Bulk RNA-seq analysis of patient-derived public datasets

Publicly available bulk RNA-seq data were reanalyzed to explore the relationship between *ZNF469* expression and ECM-associated genes in the context of pulmonary fibrosis. Specifically, RNA-seq data of lung tissue samples from IPF patients and healthy control subjects were obtained from the Gene Expression Omnibus (GEO) database (GSE92592) [33]. To extend the analysis to additional fibrotic lung contexts, transcriptomic datasets from systemic sclerosis-associated interstitial lung disease (SSc-ILD) were also retrieved and analyzed (GSE231693) [34]. Expression values were log2-transformed prior to visualization and statistical analysis. Differences in *ZNF469* and selected ECM-associated gene expression between fibrotic and control lung samples were assessed using an unpaired two-tailed Student’s *t*-test. To evaluate the association between *ZNF469* and ECM-related genes, Spearman rank correlation coefficients were calculated across samples using normalized expression values. Correlations were performed between *ZNF469* and representative fibrotic markers, including collagen type I alpha 1 chain (*COL1A1*), collagen type I alpha 2 chain (*COL1A2*), collagen type III alpha 1 chain (*COL3A1*), collagen type VI alpha 1 chain (*COL6A1*), collagen type XVI alpha 1 chain (*COL16A1*), alpha-2-macroglobulin (*A2M*), and actin alpha 2, smooth muscle (*ACTA2*). *P*-values were adjusted for multiple testing using the Benjamini-Hochberg method.

### Reanalysis of single-cell RNA-seq analysis of IPF and SSc-ILD datasets

Single-cell transcriptomic data from IPF (GSE135893) [35] and SSc-ILD (GSE128169) [36] lung samples were reanalyzed to examine *ZNF469* expression in lung-derived cell populations from healthy and disease contexts. Highly variable genes were identified using Seurat v3, followed by principal component analysis, harmony batch correction where necessary, and Uniform Manifold Approximation and Projection visualization. Cell clustering was generated using the Leiden algorithm and annotated according to canonical marker expression. Cells were categorized into four lineages: immune, epithelial, endothelial, and mesenchymal. Fibroblasts were identified by *COL1A1*, *COL3A1*, decorin (*DCN*), and lumican (*LUM*) expression. *ZNF469*-expressing fibroblasts were defined as cells with detectable expression (> 0), and high *COL1A1*-expressing fibroblasts were defined as those with *COL1A1* expression at or above the 80th percentile. Expression burden was calculated per 1,000 total cells per patient. Disease vs control comparisons were performed using a negative binomial generalized linear model with log-transformed total cells as an offset (via the MASS package in R) to account for varying cell numbers between patients. Fold changes were calculated as exp(β) with a 95% confidence interval. Spearman correlations were assessed among the genes of interest, including *ZNF469*, *COL1A1*, *COL3A1*, *COL6A1*, collagen type IX alpha 2 chain (*COL9A2*), and *COL16A1*. Benjamini-Hochberg FDR correction was applied to these correlations.

**Figure 1.**
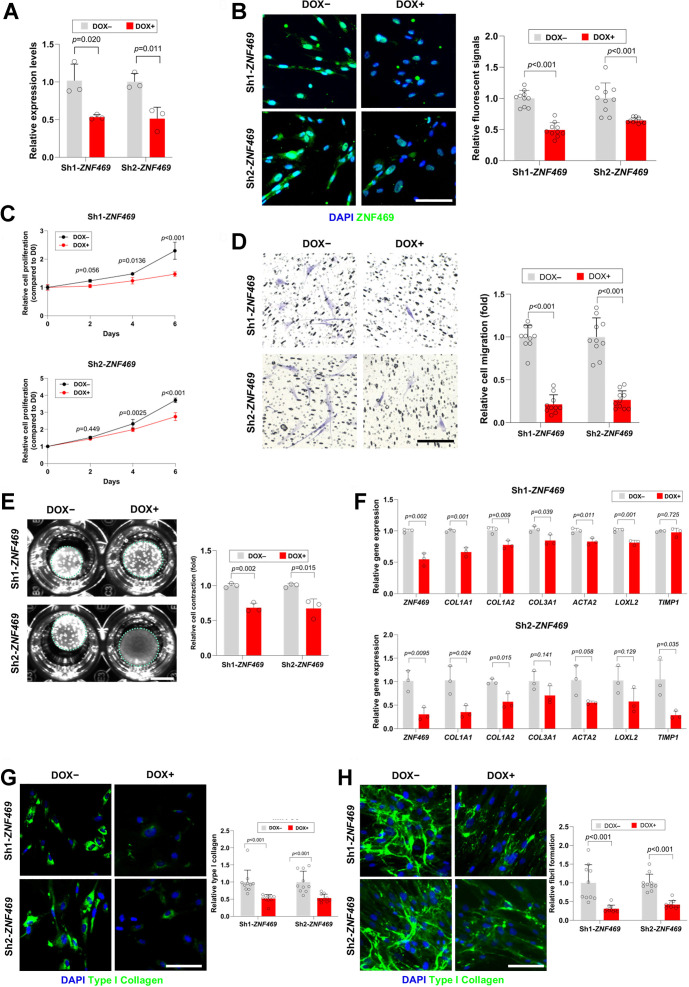
**Depletion of ZNF469 impairs activation phenotypes of human lung fibroblasts.** (A) RT-qPCR analysis of *ZNF469* mRNA expression in IMR-90 lung fibroblasts transduced with a lentiviral vector harboring a doxycycline (DOX)-inducible shRNA system targeting *ZNF469* transcripts. Non-induced cells (DOX--) served as the control group. (B) Immunofluorescence analysis of ZNF469 protein levels in control and *ZNF469*-depleted cells. Scale bar, 100 µm. (C) MTT assay quantifying cell proliferation over 6 days in control and *ZNF469*-depleted cells. (D) Transwell assay assessing the migration of control and *ZNF469*-depleted cells. Scale bar, 100 µm. (E) Collagen gel contraction assay performed with control and *ZNF469*-depleted cells. Scale bar, 0.5 cm. (F) RT-qPCR analysis of key fibrotic and activated fibroblast markers following *ZNF469* depletion. (G) Immunofluorescence staining for intracellular type I collagen following *ZNF469* depletion. Scale bar, 100 µm. (H) Immunofluorescence analysis of type I collagen fibril formation following *ZNF469* depletion. Scale bar, 100 µm. Fluorescent images, with DAPI nuclear staining in blue and type I collagen staining in green, were captured under identical exposure settings for comparative analysis. All data are presented as the mean ± SD, derived from n ≥ 3 technical replicates across two independent experiments. Statistical significance was determined using an unpaired two-tailed Student’s *t*-test (A, B, D, E–H) and two-way ANOVA with a post-hoc Šídák’s multiple comparisons test (C). Abbreviations: ANOVA, analysis of variance; DAPI, 4′,6-diamidino-2-phenylindole; DOX, doxycycline; mRNA, messenger RNA; MTT, 3-(4,5-dimethylthiazol-2-yl)-2,5-diphenyltetrazolium bromide; RT-qPCR, reverse transcription quantitative polymerase chain reaction; SD, standard deviation; shRNA, short hairpin RNA; ZNF469, zinc finger protein 469.

### Statistical analysis

Data are presented as mean ± SD. Statistical analyses were performed using GraphPad Prism (version 10.6.1; Dotmatics, Boston, MA, USA). For *in vitro* experiments, each assay included three or more technical replicates across two independent experiments. To ensure the biological generalizability of our findings, key results were validated in two distinct human lung fibroblast cell lines (IMR-90 and MRC-5). Two-group comparisons were analyzed using an unpaired two-tailed Student’s *t*-test or Mann-Whitney *U* test, as appropriate. Multi-group experiments were analyzed via one-way or two-way analysis of variance (ANOVA), followed by Tukey’s multiple-comparison test. Time-course proliferation assays were analyzed using two-way ANOVA, considering treatment and time as factors, followed by Šídák’s multiple-comparison test. Statistical significance was defined as *P* < 0.05.

### Ethical statement

This study utilized publicly available, de-identified datasets from the GEO. No new human participants were recruited; therefore, institutional review board approval and informed consent were not required.

## Results

### ZNF469 depletion impairs key activation features of lung fibroblasts

To investigate the proposed role of ZNF469 in pulmonary fibrosis, we established two independent doxycycline-inducible knockdown cell lines using IMR-90 human lung fibroblasts, enabling temporal control of *ZNF469* depletion. Efficient knockdown by both shRNAs was confirmed through RT-qPCR and immunofluorescence staining, demonstrating reduced ZNF469 expression at both the mRNA and protein levels compared with non-induced controls (Figure 1A and 1B). Functional assays indicated that ZNF469 depletion attenuated fibroblast proliferation, with modest effects observed at early time points and a more pronounced reduction at later time points (Figure 1C). Additionally, ZNF469-knockdown cells exhibited impaired migratory capacity and diminished contractile activity in collagen gel contraction assays (Figure 1D and 1E).

Consistent with these functional changes, the expression of fibroblast activation markers significantly decreased following ZNF469 knockdown. This decrease included representative ECM genes (*COL1A1*, *COL1A2*, and *COL3A1*) as well as conventional myofibroblast markers such as *ACTA2* (alpha-smooth muscle actin, α-SMA), lysyl oxidase like 2 (*LOXL2*), and TIMP metallopeptidase inhibitor 1 (*TIMP1*) (Figure 1F). Notably, while both shRNAs effectively suppressed the fibrogenic program, the magnitude of the molecular response correlated with the degree of target depletion. In RT-qPCR assays using independent cell batches, the more robust knockdown consistently achieved by sh-2 resulted in a correspondingly more pronounced reduction in fibrotic markers compared with sh-1 (Figure 1F). Conversely, for functional assays, cell batches with comparable knockdown efficiencies were utilized (Figure 1A), yielding consistent phenotypic outcomes across both independent shRNAs.

**Figure 2.**
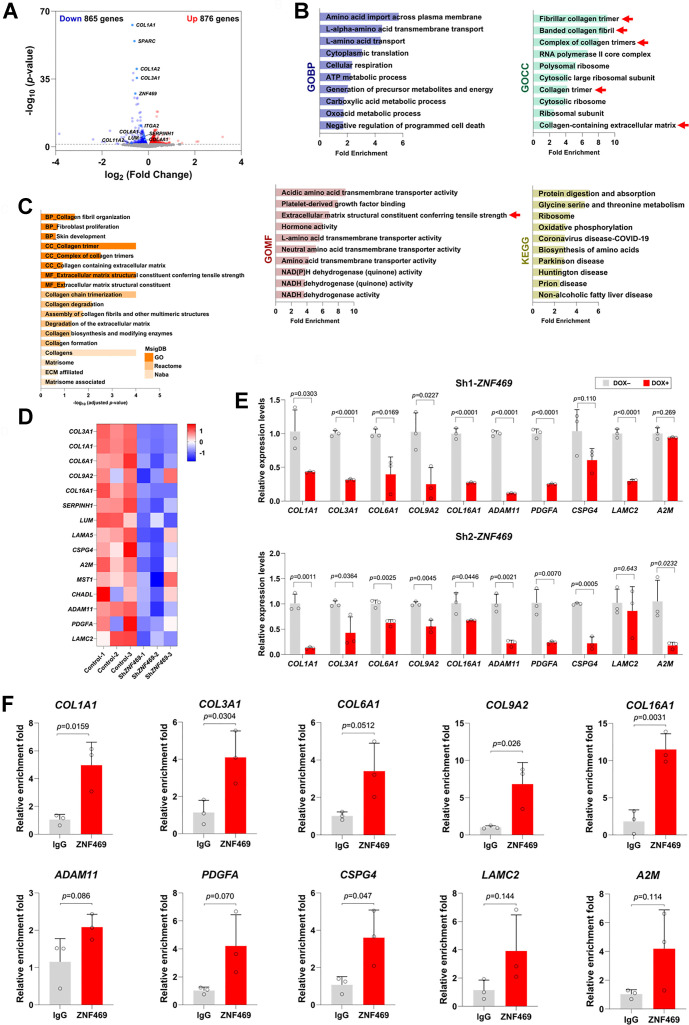
**RNA-sequencing analysis of shRNA-mediated ***ZNF469*** knockdown in human lung fibroblasts reveals impaired ECM-related pathways.** (A) Volcano plot illustrating log2 fold changes and --log10(FDR) of gene expression in *ZNF469*-knockdown IMR-90 lung fibroblasts compared to non-induced control cells. DEGs were identified using criteria of |log2 FC| > 1.0 and FDR < 0.05. (B) Functional overrepresentation analysis (KEGG and GO) of downregulated DEGs in *ZNF469*-knockdown cells relative to controls. Red arrows highlight ECM-related terms. (C) Gene Set Enrichment Analysis (GSEA) demonstrates significant downregulation of ECM- and collagen-associated pathways following *ZNF469* knockdown. (D) Heatmap illustrating representative ECM-related gene expression derived from RNA-seq analysis of control and *ZNF469*-knockdown cells. Gene expression values are presented as Z-scores, where red indicates upregulation and blue indicates downregulation (scale --1 to 1). (E) RT-qPCR validation of representative ECM-related genes following *ZNF469* knockdown, employing two independent shRNAs (sh-1 and sh-2). Non-induced cells (DOX--) served as controls. (F) qPCR analysis of ECM-related gene promoter regions following a CUT&RUN assay performed with an anti-ZNF469 antibody. Data are presented as the mean ± SD from *n* ═ 3 technical replicates. Statistical significance was determined using an unpaired, two-tailed Student’s *t*-test (E, F). Abbreviations: CUT&RUN, cleavage under targets and release using nuclease; DEG, differentially expressed gene; DOX, doxycycline; ECM, extracellular matrix; FC, fold change; FDR, false discovery rate; GO, Gene Ontology; GSEA, Gene Set Enrichment Analysis; KEGG, Kyoto Encyclopedia of Genes and Genomes; qPCR, quantitative polymerase chain reaction; RNA-seq, RNA sequencing; RT-qPCR, reverse transcription quantitative polymerase chain reaction; SD, standard deviation; shRNA, short hairpin RNA; ZNF469, zinc finger protein 469.

Immunofluorescence analysis further demonstrated reduced intracellular type I collagen production and diminished collagen fibril formation in *ZNF469*-depleted fibroblasts (Figure 1G and 1H). To confirm the broader relevance of these findings, key phenotypic and molecular changes were recapitulated in MRC-5 human lung fibroblasts (Figure S1). Additionally, control experiments confirmed that neither lentiviral transduction nor doxycycline treatment alone altered collagen production in lung fibroblasts (Figure S2). To determine whether reduced proliferation contributed to the observed functional defects, we performed mitomycin C treatment to inhibit cell division. The comparable percentage of Ki-67-positive cells between groups confirmed that proliferation was similarly suppressed across conditions (Figure S3). Even under these proliferation-restricted conditions, *ZNF469*-knockdown fibroblasts exhibited reduced migration and collagen production compared with mitomycin C-treated control cells (Figure S3). These findings suggest that the impaired fibroblast activation phenotype following *ZNF469* depletion is not solely due to reduced proliferation. Collectively, these data support *ZNF469* as a significant contributor to fibroblast activation phenotypes and ECM production in lung fibroblasts.

### Depletion of ZNF469 impairs ECM-related pathways in lung fibroblasts

To elucidate the transcriptional regulatory role of ZNF469 in lung fibroblasts, RNA-seq was conducted in control and *ZNF469*-depleted IMR-90 cells. Differential expression analysis identified 876 upregulated and 865 downregulated genes following *ZNF469* knockdown (Figure 2A). Functional overrepresentation analyses utilizing KEGG and GO revealed that ECM remodeling and collagen biosynthesis pathways were among the most significantly downregulated (Figure 2B). Consistently, gene set enrichment analysis (GSEA) confirmed a global reduction in ECM- and collagen-associated transcriptional programs in *ZNF469*-depleted cells (Figure 2C and S4). At the individual gene level, *ZNF469* depletion resulted in reduced expression of several collagen genes (*COL1A1*, *COL3A1*, *COL6A1*, *COL9A2*, and *COL16A1*), along with numerous ECM regulators, including serpin family E member 1 (*SERPINE1*), *LUM*, laminin subunit alpha 5 (*LAMA5*), and platelet derived growth factor subunit A (*PDGFA*) (Figure 2D and S4). These transcriptional changes were validated by RT-qPCR analysis of representative ECM genes (Figure 2E). To assess whether ZNF469 transcriptionally regulates these targets, CUT&RUN assays were performed to evaluate ZNF469 occupancy at ECM gene promoters. The results demonstrated robust enrichment at the promoter regions of multiple collagen genes relative to IgG controls; conversely, enrichment at other ECM gene promoters was less pronounced, suggesting that ZNF469 preferentially occupies collagen gene promoters in lung fibroblasts (Figure 2F). These findings support a role for ZNF469 in modulating ECM-associated transcriptional programs, with preferential occupancy detected at selected collagen gene promoters.

In contrast to the downregulated DEGs, which were predominantly enriched for ECM-related terms, overrepresentation analyses of the 876 upregulated DEGs revealed a distinct functional profile (Table S2). The upregulated gene set was significantly enriched for pathways related to TGF-β signaling, SMAD binding, p53 signaling, protein processing in the endoplasmic reticulum, and cell-substrate adhesion. These findings indicate that while ZNF469 depletion attenuates the fibrogenic program, it concurrently induces a separate set of transcriptional responses that may represent compensatory or alternative signaling pathways.

### ZNF469 is regulated by the canonical TGF-β1/SMAD3 axis

**Figure 3.**
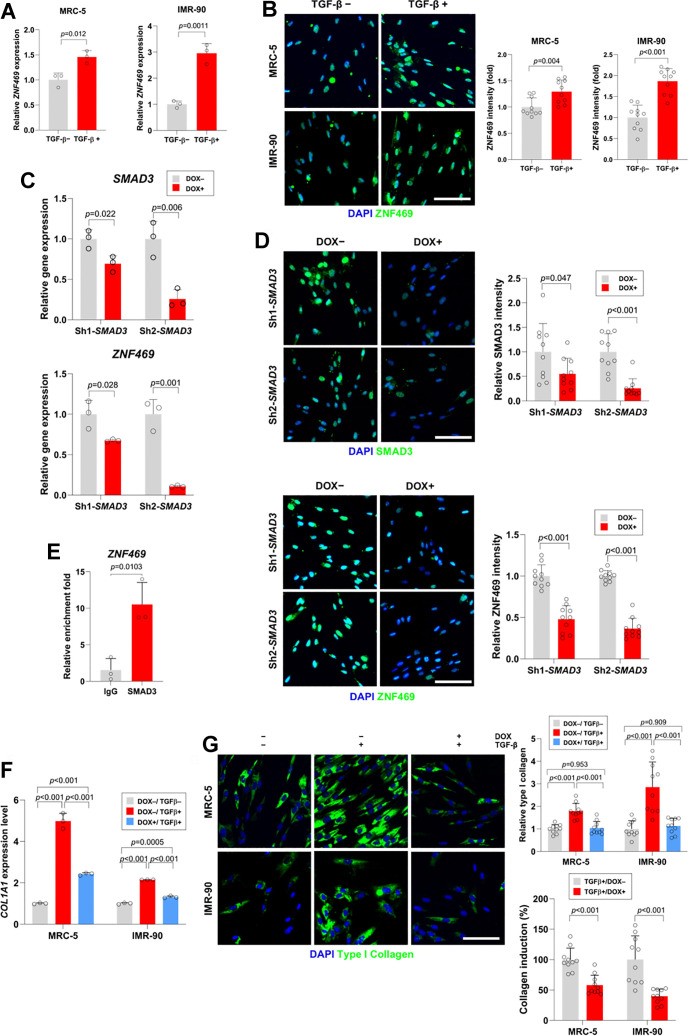
**ZNF469 is a downstream effector of the TGF-β1/SMAD3 signaling axis in human lung fibroblasts.** (A) RT-qPCR analysis of *ZNF469* mRNA levels following TGF-β1 treatment in MRC-5 and IMR-90 human lung fibroblasts. (B) Immunofluorescence detection of ZNF469 protein in human lung fibroblasts after 24-hour incubation with 10 ng/mL TGF-β1. Scale bar, 100 µm. (C) RT-qPCR analysis of *SMAD3* and *ZNF469* mRNA levels in IMR-90 human lung fibroblasts transduced with a doxycycline-inducible lentiviral system expressing shRNAs targeting *SMAD3* mRNA. Non-induced cells (DOX--), lacking doxycycline, served as the control group. (D) Immunofluorescence detection of SMAD3 and ZNF469 proteins in control and *SMAD3*-knockdown IMR-90 human lung fibroblasts. Scale bar, 100 µm. (E) qPCR analysis of *ZNF469* gene promoter regions after a CUT&RUN assay performed with an anti-SMAD3 antibody. (F) RT-qPCR validation of *COL1A1* expression in *ZNF469*-depleted human lung fibroblasts following TGF-β1 treatment. (G) Immunofluorescence detection of type I collagen in *ZNF469*-depleted human lung fibroblasts after TGF-β1 treatment. Quantification of residual collagen induction following ZNF469 depletion was calculated by normalizing the TGF-β1-treated/DOX-- condition to 100% to assess the relative degree of suppression. Scale bar, 100 µm. Data are presented as the mean ± SD of n ≥ 3 technical replicates from two independent experiments. Statistical significance was determined using an unpaired two-tailed Student’s *t*-test (A-E) and two-way ANOVA with a post-hoc Tukey (F, G). Abbreviations: ANOVA, analysis of variance; COL1A1, collagen type I alpha 1 chain; CUT&RUN, cleavage under targets and release using nuclease; DOX, doxycycline; mRNA, messenger RNA; qPCR, quantitative polymerase chain reaction; RT-qPCR, reverse transcription quantitative polymerase chain reaction; SD, standard deviation; shRNA, short hairpin RNA; SMAD3, SMAD family member 3; TGF-β1, transforming growth factor beta 1; ZNF469, zinc finger protein 469.

TGF-β1 is a canonical profibrotic cytokine that drives fibroblast activation and ECM accumulation in fibrotic diseases, including IPF [37, 38]. Previous evidence indicated that ZNF469 is transcriptionally regulated downstream of SMAD3 following short-term stimulation with TGF-β1 in hepatic stellate cells [20]. To ascertain whether this TGF-β1/SMAD3/ZNF469 regulatory axis operates in lung fibroblasts, we first examined ZNF469 expression following TGF-β1 treatment in IMR-90 and MRC-5 cells. TGF-β1 stimulation significantly increased ZNF469 expression at both mRNA and protein levels (Figure 3A and 3B).

To assess whether this induction depends on SMAD3, we generated two shRNA-inducible lines of IMR-90 cells targeting *SMAD3* transcripts. Efficient knockdown by both independent shRNAs was confirmed by RT-qPCR and immunofluorescence (Figure 3C and 3D). Notably, ZNF469 expression markedly decreased following SMAD3 knockdown, indicating that ZNF469 is a downstream target of SMAD3 in lung fibroblasts (Figure 3C and 3D). The suppression of *ZNF469* transcript levels correlated with the potency of *SMAD3* depletion; the more robust *SMAD3* knockdown achieved by sh-2 resulted in a correspondingly more pronounced reduction in ZNF469 expression compared with sh-1 (Figure 3C). This apparent dose-dependent relationship reinforces the hierarchical link between these factors. Furthermore, CUT&RUN analysis revealed SMAD3 enrichment at the *ZNF469* promoter, supporting a direct transcriptional regulatory mechanism (Figure 3E).

To evaluate the functional necessity of ZNF469 in the TGF-β1 response, we assessed collagen expression following TGF-β1 stimulation in the context of *ZNF469* depletion. ZNF469 knockdown markedly attenuated, but did not completely abolish, TGF-β1-mediated collagen production (Figure 3F and 3G). To further evaluate the degree to which the profibrotic response depends on ZNF469, we quantified the residual collagen induction following TGF-β1 stimulation in *ZNF469*-depleted cells. By normalizing the TGF-β1-induced response in non-induced cells (DOX--) to 100%, we determined that *ZNF469* depletion blunted collagen production by approximately 40%–50% in both MRC-5 and IMR-90 fibroblasts (Figure 3G). These data indicate that while ZNF469 is a downstream effector of the canonical TGF-β1 pathway, a significant portion of the collagen response (approximately 50%–60%) persists upon its depletion. This residual induction suggests the involvement of parallel, ZNF469-independent signaling mechanisms in the regulation of ECM genes.

### ZNF469 upregulation correlates with fibrotic gene signatures in human IPF and SSc-ILD

**Figure 4.**
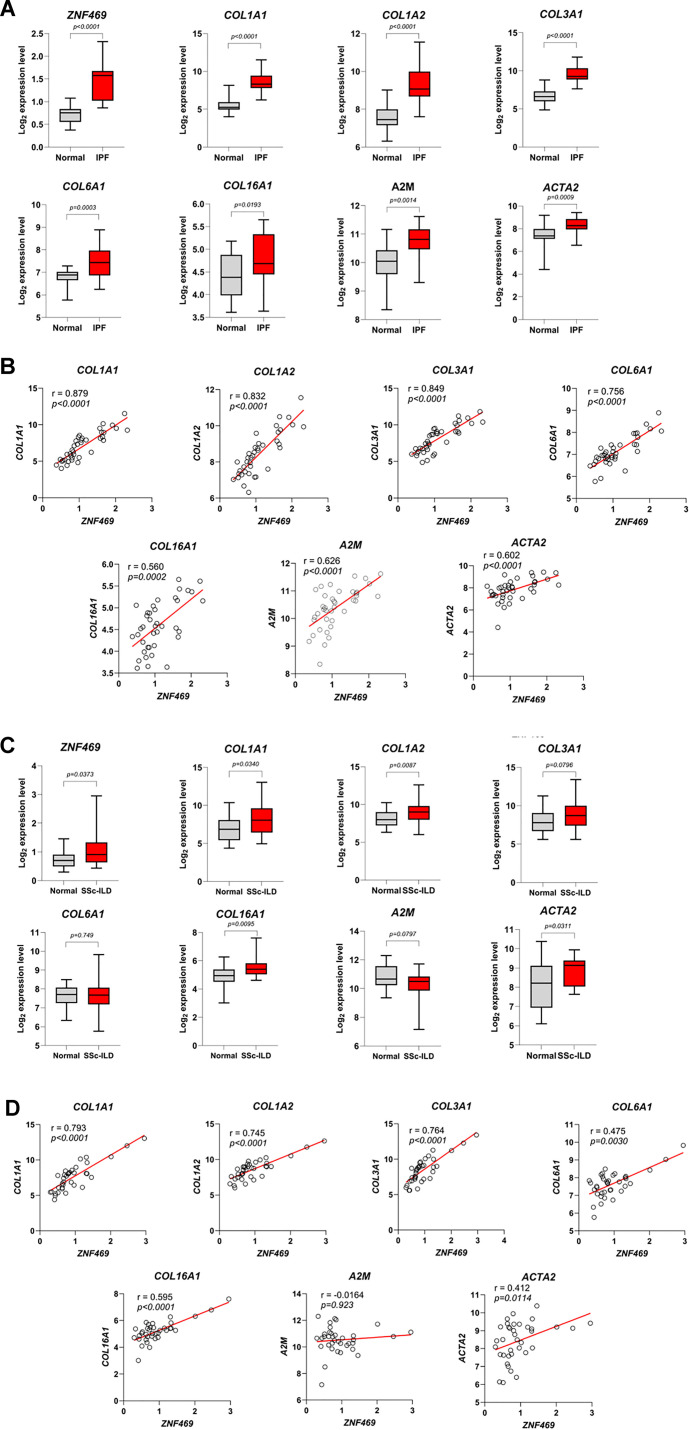
*****ZNF469*** is upregulated in human lung fibrotic tissues and correlates with ECM signatures.** (A) Reanalysis of RNA-seq data from the GSE92592 dataset presented as box-and-whisker plots showing *ZNF469* and ECM-related gene expression in normal (*n* ═ 19) and IPF tissues (*n* ═ 20). (B) Spearman correlation analysis between *ZNF469* expression and ECM-related gene expression in normal and IPF samples. (C) Reanalysis of RNA-seq data from the GSE231693 dataset showing *ZNF469* and ECM-related gene expression in normal (*n* ═ 18) and SSc-ILD tissues (*n* ═ 19). (D) Spearman correlation analysis between *ZNF469* expression and ECM-related gene expression in normal and SSc-ILD samples. Statistical significance was determined using an unpaired two-tailed Student’s *t*-test (A, C). Correlations were assessed using Spearman rank correlation (B, D). Abbreviations: ECM, extracellular matrix; IPF, idiopathic pulmonary fibrosis; RNA-seq, RNA sequencing; SSc-ILD, systemic sclerosis-associated interstitial lung disease; ZNF469, zinc finger protein 469.

To evaluate the clinical relevance of *ZNF469* in fibrotic lung diseases, we reanalyzed publicly available bulk RNA-seq datasets. Analysis of the GSE92592 IPF cohort revealed significant upregulation of *ZNF469* expression in fibrotic lungs compared to normal controls, accompanied by increased expression of multiple ECM genes (Figure 4A). Correlation analysis showed positive associations between *ZNF469* and canonical fibrotic markers, including *COL1A1*, *COL1A2*, *COL3A1*, *COL6A1*, *COL16A1*, *A2M*, and *ACTA2* (Figure 4B), further supporting a relationship between ZNF469 expression and fibrotic gene signatures. Similar trends were observed in the GSE231693 SSc-ILD datasets, where *ZNF469* and several ECM-related genes were significantly elevated compared to normal lung tissues (Figure 4C and 4D). Given the distinct clinical origins of both IPF and SSc-ILD [39, 40], these findings suggest that *ZNF469* upregulation represents a shared transcriptional feature of fibroblast activation and ECM remodeling across diverse fibrotic contexts, rather than being restricted to a specific disease etiology.

### Single-cell RNA-seq reveals ZNF469 enrichment in activated fibroblast populations in fibrotic lungs

**Figure 5.**
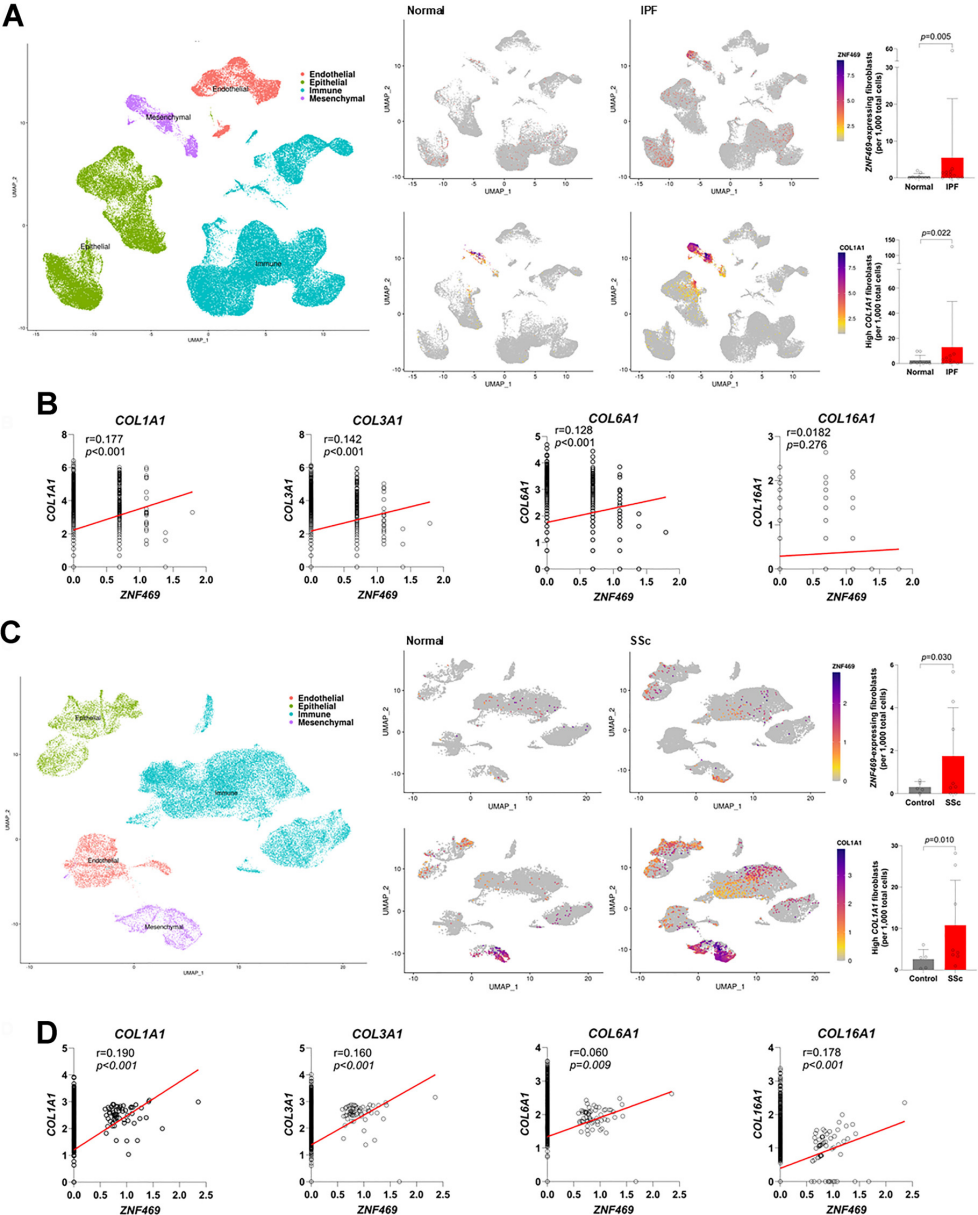
**Single-cell analysis of lung fibrotic tissues reveals upregulation of ***ZNF469*** in activated lung fibroblasts.** (A) UMAP plots illustrating lung-derived cell populations and the spatial distribution of *ZNF469* (upper panel) and *COL1A1* (lower panel) expression in normal (*n* ═ 9) and Idiopathic Pulmonary Fibrosis (IPF) (*n* ═ 12) samples from the GSE135893 dataset. Expression levels are indicated by a color gradient from grey (low) to purple (high) on a scale of 2.5 to 7.5. Bar graphs quantify the proportion of *ZNF469*-positive and high *COL1A1*-expressing fibroblasts in normal and IPF samples. (B) Spearman correlation analysis between *ZNF469* and collagen-related genes, derived from single-cell data in normal and IPF samples. (C) UMAP plots illustrating lung-derived cell populations and the spatial distribution of *ZNF469* (upper panel) and *COL1A1* (lower panel) expression in normal (*n* ═ 6) and Systemic Sclerosis-associated Interstitial Lung Disease (SSc-ILD) (*n* ═ 8) samples from the GSE128169 dataset. Expression levels are indicated by a color gradient from grey (low) to purple (high) on a scale of 0.0 to 2.0. Bar graphs quantify the proportion of *ZNF469*-positive and high *COL1A1*-expressing fibroblasts in normal and SSc-ILD samples. (D) Spearman correlation analysis between *ZNF469* and collagen-related genes, derived from single-cell data in normal and SSc-ILD samples. Statistical significance was determined using the Mann-Whitney *U* test (A, C). Correlations were assessed using Spearman rank correlation (B, D). Abbreviations: COL1A1, collagen type I alpha 1 chain; IPF, idiopathic pulmonary fibrosis; SSc-ILD, systemic sclerosis-associated interstitial lung disease; UMAP, Uniform Manifold Approximation and Projection; ZNF469, zinc finger protein 469.

To define the cellular distribution of *ZNF469* at single-cell resolution, we reanalyzed the single-cell RNA-seq dataset (GSE135893), comprising single-cell suspensions from healthy control and IPF lungs. Analysis of mesenchymal clusters revealed that *ZNF469*, alongside collagen genes, was markedly enriched in fibroblast populations within IPF lungs compared to controls (Figure 5A and S5). Notably, the proportion of *ZNF469*-positive fibroblasts significantly expanded in the fibrotic state, specifically within clusters exhibiting high *COL1A1* expression. Correlation analysis at the single-cell level further supported a positive association between *ZNF469* and transcript levels of core collagen genes, including *COL1A1* and *COL3A1* (Figure 5B). This association varied across the broader ECM repertoire; for instance, the correlation with *COL16A1* did not reach statistical significance in the IPF dataset, suggesting that ZNF469 may preferentially coordinate specific subsets of the fibrogenic program rather than the entire matrisome.

To determine whether this signature is conserved across distinct fibrotic etiologies, we analyzed the SSc-ILD dataset (GSE128169). Consistent with findings in IPF, *ZNF469* expression was prominently localized to the mesenchymal compartment in SSc-ILD lungs. While *ZNF469* transcripts were detectable in other cell types, the proportion of *ZNF469*-positive fibroblasts was significantly increased in fibrotic lungs relative to healthy controls (Figure 5C). Furthermore, single-cell correlation analysis in the SSc-ILD cohort displayed a positive relationship between *ZNF469* and collagen gene expression (Figure 5D). Collectively, these single-cell analyses indicate that *ZNF469* upregulation is associated with a conserved feature of activated fibroblast populations in both IPF and SSc-ILD, underscoring the clinical relevance of ZNF469 in human lung diseases.

## Discussion

In this study, we identify ZNF469 as a previously unrecognized yet key regulator of the fibrogenic program in human lung fibroblasts. Utilizing independent inducible knockdown models in IMR-90 and MRC-5 cells, we demonstrate that *ZNF469* depletion consistently attenuates key activated fibroblast phenotypes, including proliferation, migration, contractility, and collagen production. Notably, we establish that these phenotypic changes are not merely secondary consequences of reduced cell growth, as the impairment of collagen synthesis and migration remained evident even when cell proliferation was pharmacologically inhibited with mitomycin C.

Integrated transcriptomic profiling and CUT&RUN analysis further support a role for ZNF469 in regulating ECM-associated transcriptional programs, with ZNF469 enrichment detected at the promoters of core collagen genes, including *COL1A1* and *COL3A1*. However, the comparatively weaker occupancy observed at other non-collagen ECM loci suggests broader regulatory involvement that warrants further research. Our findings also reveal that *ZNF469* expression is modulated by the TGF-β1/SMAD3 signaling axis, supporting a model in which ZNF469 functions as a downstream mediator of specific profibrotic signaling outputs in lung fibroblasts. The clinical relevance of this model is reinforced by our reanalysis of bulk and single-cell RNA-seq data from patients with IPF and SSc-ILD, which show *ZNF469* upregulation and enrichment within collagen-producing fibroblast populations. These findings suggest that ZNF469 is a significant factor contributing to fibroblast-mediated matrix remodeling across diverse interstitial lung diseases.

The involvement of *ZNF469* in profibrotic gene regulation appears to be a conserved feature across multiple mesenchymal cell contexts. In hepatic stellate cells, previous studies have demonstrated that *ZNF469* silencing reduces proliferation, migration, and collagen production [20]. These phenotypic changes are mirrored by transcriptomic shifts in ECM-related pathways and occupancy of ZNF469 at the *COL1A1* and *COL1A2* promoters, as shown by chromatin immunoprecipitation [20]. Furthermore, a recent unbiased clustered regularly interspaced short palindromic repeats (CRISPR) loss-of-function screen identified ZNF469 as the top-ranking transcriptional regulator of *COL1A1* expression [22]. Notably, CRISPR-mediated knockout of *ZNF469* in LX-2 hepatic stellate cells led to significantly decreased histone H3 lysine 27 acetylation (H3K27ac) enrichment at the *COL1A1* and *COL1A2* loci, highlighting its specific role in controlling type I collagen gene activation [22]. Consistent findings were also reported in dermal fibroblasts, where *ZNF469* silencing impaired fibroblast proliferation and ECM production while confirming its binding to promoters of collagen-related genes [21]. By extending these observations to lung fibroblasts, our study suggests that ZNF469 participates in a conserved profibrotic transcriptional program across diverse organ systems. However, the degree of functional equivalence across different anatomical sites and disease contexts remains to be fully defined.

Mechanistically, whether ZNF469 regulates gene expression through sequence-specific DNA binding or via recruitment to chromatin-associated complexes remains to be fully elucidated. Given that ZNF469 contains multiple predicted Cys2His2 zinc-finger domains [17], which are characteristic of DNA-binding proteins, it is plausible that it recognizes specific genomic motifs or interacts with transcriptional co-regulators to modulate the chromatin landscape [41, 42]. Future studies will be required to define the DNA-binding properties of ZNF469 and identify its protein-interacting partners, which will be essential to delineate how these molecular interactions coordinate the fibrogenic program in activated fibroblasts.

Beyond its association with ECM gene regulation, our data support ZNF469 as a putative downstream mediator of the canonical TGF-β/SMAD3 signaling pathway in lung fibroblasts. While TGF-β is the most potent driver of fibrogenesis, clinical efforts to target the cytokine directly have often been hindered by its pleiotropic roles in immune regulation, physiological homeostasis, and tumor suppression [43, 44]. Consequently, systemic blockade of TGF-β signaling may not provide a selective antifibrotic benefit without perturbing essential physiological processes [45, 46]. Identifying specific downstream mediators that relay profibrotic signals while sparing non-pathological functions remains a primary therapeutic goal. Unlike SMAD3, which regulates thousands of genes across diverse cellular processes [47, 48], ZNF469 appears to have a more restricted repertoire primarily focused on ECM assembly and fibroblast activation. This specificity positions ZNF469 as a potentially advantageous therapeutic target, offering a means to decouple the pathological fibrotic response from the broader, essential physiological functions of the TGF-β superfamily.

Our experimental findings provide mechanistic support for this hierarchical positioning of ZNF469. We demonstrate that SMAD3 is enriched at the *ZNF469* promoter, and crucially, the observation that ZNF469 depletion reduces TGF-β1-induced collagen production by 40%–50% quantifies its contribution to the fibrogenic program. This partial attenuation is consistent with the complexity of TGF-β1 signaling, which engages a broad network of transcriptional and non-transcriptional effectors. The residual response observed in our models likely reflects the activity of ZNF469-independent pathways, such as the mitogen-activated protein kinase (MAPK), epidermal growth factor receptor (EGFR), or Wnt/β-catenin axes, which can independently drive collagen expression [49–51]. Recognizing ZNF469 as a significant, but not exclusive, effector for TGF-β1 signals allows for a more nuanced understanding of its therapeutic potential, suggesting that its inhibition could meaningfully attenuate matrix production while potentially sparing other homeostatic pathways.

Notably, the overrepresentation analysis of genes upregulated following ZNF469 knockdown revealed an enrichment of pathways related to TGF-β signaling, SMAD binding, p53 signaling, and proteostatic stress responses. These upregulated programs likely reflect secondary or adaptive responses to impaired ECM production or altered metabolic and proteostatic demands within fibroblasts. This interpretation is consistent with the extensive documentation of disruption or structural defects in core ECM components triggering compensatory cellular feedback loops. For instance, mouse models of osteogenesis imperfecta, characterized by defective type I collagen matrix assembly, exhibit significant hyperactivation of the TGF-β pathway and increased expression of downstream target genes due to altered matrix-cell signaling [52]. Similarly, transcriptomic profiling of collagen VI-deficient models revealed comparable activation of TGF-β-related transcriptional programs, including the upregulation of SMAD3, SMAD family member 7 (SMAD7), and tumor protein p53 (TP53) [53]. Moreover, we cannot exclude the possibility that ZNF469 functions as a transcriptional repressor at certain loci, either through direct DNA binding or the recruitment of repressive co-regulators. While our CUT&RUN data primarily support its role as an activator of collagen genes, future studies aimed at identifying the full repertoire of ZNF469-interacting partners and its specific DNA-binding motifs will be essential to determine whether it exerts dual regulatory roles as both an activator and a repressor depending on the genomic context.

The clinical significance of the *ZNF469* axis is reinforced by our reanalysis of patient-derived datasets, which suggest that *ZNF469* upregulation is associated with a common feature of human fibrotic lung disease. Although IPF and SSc-ILD possess distinct pathogenic origins—primarily driven by epithelial-mesenchymal crosstalk and systemic autoimmunity, respectively—our analysis indicated elevated *ZNF469* expression in both contexts. At the single-cell resolution, we observed that *ZNF469* is not merely upregulated across the entire tissue but is specifically enriched and proportionally expanded within activated fibroblast subpopulations. These *ZNF469*-positive fibroblasts exhibited coordinated expression of core collagen genes, suggesting that *ZNF469* marks a fibroblast population transcriptionally primed for high-output matrix production. Interestingly, recent single-cell analyses of keloid fibroblasts similarly localized *ZNF469* to a key mesenchymal subpopulation, with pseudotime analysis suggesting its pivotal role in lineage commitment and maturation of the collagen-producing phenotype [21, 54]. The conservation of this signature across multiple organs and etiologies indicates that ZNF469 may represent a common terminal pathway in fibrogenesis. Consequently, targeting ZNF469 could offer a broad therapeutic strategy for diverse fibroproliferative diseases by addressing the convergent fibrotic responses across different disease etiologies.

Despite the consistency of our findings, several limitations of this study warrant consideration. First, our mechanistic insights are primarily derived from *in vitro* human lung fibroblast models and the reanalysis of existing clinical datasets; therefore, future studies necessitate *in vivo* validation. As *ZNF469*-mutant mouse models are not currently commercially available, generating custom-engineered models, although resource-intensive, is necessary to confirm the systemic therapeutic efficacy and safety of *ZNF469* inhibition in a complex physiological environment.

Second, while protein-level validation was primarily based on immunofluorescence, the absence of complementary Western blot analysis represents a limitation of the current study. Immunofluorescence was prioritized because it uniquely allows for the visualization of ECM organization and collagen fiber assembly, key functional readouts of fibroblast activation that are not captured in bulk cell lysates. Furthermore, this approach enabled the confirmation of ZNF469’s nuclear localization, consistent with its predicted role as a transcriptional effector. We also note that the specific antibodies utilized are optimized for immunostaining rather than biochemical detection under denaturing conditions [22, 55–60]. Future investigations employing Western-optimized reagents or endogenous protein-tagging systems will be instrumental in providing additional biochemical quantification of ZNF469 expression and its broader signaling interactions.

Third, the absence of complementary gain-of-function or rescue experiments is a notable limitation of this study. Validating the specificity of the observed effects through the re-expression of ZNF469 in knockdown cells is technically challenging due to the exceptionally large size of its coding sequence (∼12 kb), which exceeds the packaging capacity of standard lentiviral vectors and hampers efficient transfection. To mitigate the potential for off-target effects in the absence of rescue data, we utilized two independent shRNA sequences for all functional and molecular experiments, yielding consistent and reproducible outcomes across two lung fibroblast cell lines. Developing modular or truncated ZNF469 systems for functional rescue will be a critical objective for future research. Finally, while our data establish ZNF469 as a key regulator of collagen gene expression, the precise biochemical mechanisms governing its recruitment—specifically whether it involves sequence-specific DNA binding or indirect recruitment via chromatin-associated co-factors—remain to be fully elucidated. Identifying the protein-interacting partners of ZNF469 will be essential for mapping the complete hierarchical structure of this profibrotic transcriptional program.

## Conclusion

In summary, our study establishes ZNF469 as a key transcriptional regulator of lung fibroblast activation and ECM production. We demonstrate that ZNF469 is a downstream target of the TGF-β1/SMAD3 signaling axis and preferentially occupies core collagen gene promoters. Functional depletion of ZNF469 is sufficient to attenuate the profibrotic effects of TGF-β1, characterizing it as a significant effector of this canonical pathway. These mechanistic insights are corroborated by clinical transcriptomic analyses across IPF and SSc-ILD, where ZNF469 expression is specifically enriched and expanded within activated fibroblast subpopulations. Collectively, our findings position ZNF469 as a conserved and critical component of the profibrotic regulatory machinery in lung fibroblasts, highlighting its potential as a promising candidate for the development of targeted therapies to mitigate fibroproliferative lung diseases.

**AI declaration statement:** During the preparation of this work, ChatGPT, an artificial intelligence tool, was used to improve the readability and language of the manuscript. Subsequently, the authors revised and edited the content produced by the artificial intelligence tool as necessary, taking full responsibility for the ultimate content of the present manuscript.

## Supplemental data

Supplemental data are available at the following link: https://www.bjbms.org/ojs/index.php/bjbms/article/view/14165/4209

## Data Availability

The RNA-seq data sets generated in the present study may be found in the National Center for Biotechnology Information Gene Expression Omnibus repository under accession number GSE318606. All other data generated in the present study may be requested from the corresponding author.
